# High Prevalence and Diversity of Cephalosporin-Resistant *Enterobacteriaceae* Including Extraintestinal Pathogenic *E. coli* CC648 Lineage in Rural and Urban Dogs in Northwest Spain

**DOI:** 10.3390/antibiotics9080468

**Published:** 2020-08-01

**Authors:** Fátima Abreu-Salinas, Dafne Díaz-Jiménez, Isidro García-Meniño, Pilar Lumbreras, Ana María López-Beceiro, Luis Eusebio Fidalgo, María Rosario Rodicio, Azucena Mora, Javier Fernández

**Affiliations:** 1 Servicio de Microbiología, Hospital Universitario Central de Asturias, 33011 Oviedo, Spain; fatimabreu2688@gmail.com (F.A.-S.); pilar.lumbreras08@gmail.com (P.L.); 2 Grupo de Microbiología Traslacional, Instituto de Investigación Sanitaria del Principado de Asturias (ISPA), 33011 Oviedo, Spain; rrodicio@uniovi.es; 3 Laboratorio de Referencia de *Escherichia coli* (LREC), Dpto. de Microbioloxía e Parasitoloxía, Universidade de Santiago de Compostela (USC), 15705 Lugo, Spain; dafne.diaz@usc.es (D.D.-J.); isidro.garcia@usc.es (I.G.-M.); azucena.mora@usc.es (A.M.); 4 Instituto de Investigación Sanitaria de Santiago de Compostela (IDIS), 15706 Santiago, Spain; 5 Dpto. de Anatomía, Produción Animal e Ciencias Clínicas Veterinarias, Universidade de Santiago de Compostela (USC), 15705 Lugo, Spain; anam.lopez.beceiro@usc.es (A.M.L.-B.); luis.fidalgo@usc.es (L.E.F.); 6 Departamento de Biología Funcional (Área de Microbiología), Universidad de Oviedo, 33003 Oviedo, Spain

**Keywords:** ST1485 (CC648), ExPEC, MDR, ESBL, AmpC, dogs, *E. coli*, *K. pneumoniae*

## Abstract

The aim of this work was to assess the prevalence of extended spectrum-β-lactamase (ESBL)- and carbapenemase-producing *Enterobacteriaceae* in fecal samples recovered from rural and urban healthy dogs in Northwest Spain (Galicia) to identify potential high-risk clones and to molecularly characterize positive isolates regarding the genes coding for ESBL/pAmpC resistance and virulence. Thirty-five (19.6%) out of 179 dogs were positive for cephalosporin-resistant *Enterobacteriaceae*, including *Escherichia* *coli* and *Klebsiella pneumoniae* (39 and three isolates, respectively). All the isolates were multidrug resistant, with high rates of resistance to different drugs, including ciprofloxacin (71.4%). A wide diversity of ESBL/pAmpC enzymes, as well as *E. coli* phylogroups (A, B1, C, D, E, F and clade I) were found. The eight isolates (20.5%) found to conform to the ExPEC status, belonged to clones O1:H45-clade I-ST770 (CH11-552), O18:H11-A-ST93-CC168 (CH11-neg), O23:H16-B1-ST453-CC86 (CH6-31), and O83:H42-F-ST1485-CC648 (CH231-58), with the latter also complying the uropathogenic (UPEC) status. The three *K. pneumoniae* recovered produced CTX-M-15 and belonged to the ST307, a clone previously reported in human clinical isolates. Our study highlights the potential role of both rural and urban dogs as a reservoir of high-risk *Enterobacteriaceae* clones, such as the CC648 of *E. coli* and antimicrobial resistance traits. Within a One-Health approach, their surveillance should be a priority in the fight against antimicrobial resistance.

## 1. Introduction

The increase of antibiotic resistance represents a global threat to human and animal health, being that therapeutic options to combat infections have drastically reduced in recent years [1,2]. According to the list of antibiotic-resistant “priority pathogens” of the World Health Organization (WHO), multidrug-resistant (MDR) *Enterobacteriaceae*, and specifically those producing extended-spectrum β-lactamases (ESBL) and/or resistant to carbapenems, are critical pathogens in hospitals [2]. Within the *Enterobacteriaceae* family, *Klebsiella pneumoniae* and *Escherichia coli* are currently among the most important clinical burdens for human and animal health, since they have developed resistance against antibiotics regarded as the last line of defense against MDR bacteria [1]. *E. coli* is a common member of the intestinal microbiota of humans and other mammals, including dogs. However, *E. coli* can also act as a pathogen causing a wide range of infections from enteric to extraintestinal diseases, which defines the two main pathogenic categories: diarrheagenic *E. coli* (DEC) and extraintestinal pathogenic *E. coli* (ExPEC) [3,4]. ExPEC include a heterogeneous group defined by isolation from locations outside the intestinal tract with no set of genes able to unequivocally distinguish them from commensal *E. coli*: avian pathogenic *E. coli* (APEC), neonatal meningitis *E. coli* (NMEC), and uropathogenic *E. coli* (UPEC) [3,5]. In fact, they can colonize the intestinal tract, which in turn can serve as reservoir [5]. It is particularly within the group of ExPEC, where the successful high-risk clones of *E. coli* emerge [6]. The constant antibiotic selective pressure in human and animal health may contribute to the selection and further transmission of antibiotic-resistant bacteria from animals to humans and vice versa [7,8]. Considering that the number of humans having pets is increasing worldwide, companion animals may play an important role in the dissemination of MDR bacteria to their owners [9,10,11]. Currently, numerous studies report on the colonization and infection of companion animals by MDR bacteria, including ESBL- and carbapenemase-producing *Enterobacteriaceae* of a wide variety of species and clones, with a wide range of enzymes [10,11,12,13,14]. These bacteria may be acquired via different routes, such as direct contact with humans, feeding with raw food products, and outdoor living or interacting [15,16,17].

Institutions, such as the World Health Organization (WHO), the European Centre for Disease Prevention and Control (ECDC), and the Centers for Disease Control and Prevention (CDC), urge an effort to implement a “One-Health” approach involving human and veterinary health collaboration to fight against antibiotic resistance [18,19]. For this reason, the aim of this work was to assess the prevalence of ESBL- and carbapenemase-producing *Enterobacteriaceae* in fecal samples of rural and urban healthy dogs in Northwest Spain (Galicia), to identify potential high-risk clones for humans and to molecularly characterize positive isolates regarding the genes coding for ESBL/pAmpC (plasmid-mediated AmpC-beta-lactamases) resistance and virulence. 

## 2. Results

Thirty-five (19.6%) of the 179 dogs tested carried cephalosporin-resistant *Enterobacteriaceae* (10 from urban and 25 from rural environments, 20.8% and 19.1%, respectively). From the 35 positive dogs, 39 ESBL- and/or pAmpC-producing *E. coli* and three *K. pneumoniae* ESBL-producing isolates were recovered. Six dogs (14.3%) carried more than one ESBL- and/or AmpC-producing *Enterobacteriaceae* (Table S1): *E. coli* and *K. pneumoniae* (three dogs), or two and three different *E. coli* isolates (two and a single dog, respectively). 

All 42 isolates were MDR, defined as non-susceptible to at least one agent in three or more antimicrobial categories according to the Magiorakos criteria [20]. Specifically, all isolates (100%) were resistant to ampicillin, cefuroxime, and cefotaxime; 30 to ciprofloxacin (71.4%); 22 to trimethoprim/sulfamethoxazole (52.4%); 15 to tobramycin (35.7%); 13 to gentamycin (31.0%); 12 to amoxicillin/clavulanic acid (28.6%); six to amikacin (14.3%); and a single one to piperacillin/tazobactam (2.4%). Resistance to carbapenems, tigecycline, and colistin was not detected, and all isolates were negative for the *mcr* genes (1 to 5) analyzed.

**Figure 1:**
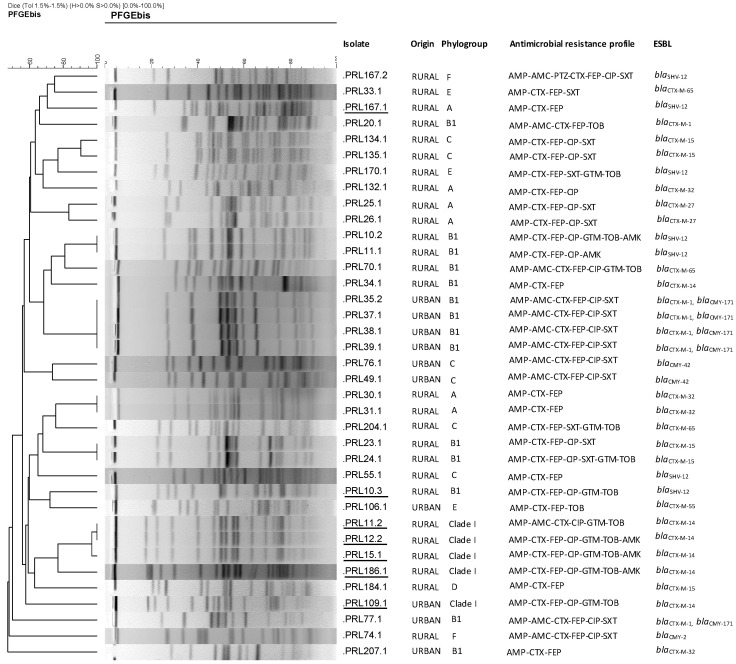
Dendrogram of genomic similarity obtained by XbaI-PFGE typing of ESBL-producing *Escherichia coli* isolates from rural and urban dogs in Northwest Spain. Isolate numeration was done by using the correlative number of the dog studied, followed by a dot and a second number if the same dog has more than one positive isolate. AMP, ampicillin; AMC, amoxicillin/clavulanic acid; PTZ, piperacillin/tazobactam; CTX, cefotaxime; FEP, cefepime; CIP, ciprofloxacin; SXT, trimethoprim/sulphamethoxazole; GTN, gentamicin; TOB, tobramycin; AMK, amikacin. Isolates conforming ExPEC status are underlined. Two isolates (PRL10.1, which also fulfills the ExPEC status, and PRL19.1) are not shown since they were non-typable by PFGE due to DNA degradation.

Among the 36 ESBL *E. coli* producers, 27 (75%) carried a *bla*_CTX-M_ gene: *bla*_CTX-M-1_ (6), *bla*_CTX-M-14_ (6), *bla*_CTX-M-15_ (5), *bla*_CTX-M-32_ (4), *bla*_CTX-M-65_ (3), *bla*_CTX-M-27_ (2), *bla*_CTX-M-55_ (1), while nine (25%) were positive for *bla*_SHV-12_. In addition, eight *E. coli*, each from a different dog, were positive for a pAmpC-encoding gene *bla*_CMY-2-like_: *bla*_CMY-171_ (5), *bla*_CMY-42_ (2) and *bla*_CMY-2_ (1). The phylogenetic analysis of the 39 broad-spectrum cephalosporin-resistant *E. coli* (ESBL/pAmpC producers) revealed a high heterogeneity, with isolate belonging to six phylogroups *sensu stricto* (A, B1, C, D, E and F) and clade I. The phylogroup B1 was the most prevalent (14 isolates; 35.9%), followed by A (7), C (6), clade I (5), E (3), F (3), and D (1). The screening of virulence traits showed that eight (20.5%) of *E. coli* isolates conformed the ExPEC status and belonged to four clones (combination of serotype-phylogroup-ST/CC-clonotype): O1:H45-clade I-ST770 (CH116-552) (5 isolates), O18:H11-A-ST93-CC168 (CH11-neg) (1 isolate), O23:H16-B1-ST453-CC86 (CH6-31) (1 isolate), and O83:H42-F-ST1485-CC648 (CH231-58) (1 isolate). The latter, (ST1485-CC648), also complied with the UPEC status. These isolates carried additional extraintestinal virulence traits (Table 1). Of note is the high number of virulence genes (16) of the ST1485-CC648, SHV-12-producing isolate. Pulse field gel electrophoresis (PFGE) performed to all *E. coli* isolates yielded 30 profiles (Figure 1). 

The XbaI-PFGE dendrogram corroborated their diversity with some isolates showing less than 60% similarity. However, six clusters (>85% identity) grouped individual isolates belonging to the same phylogroup and recovered in the same location (Figure 1; Table S1). Two isolates were non-typable by PFGE due to DNA degradation: PRL19.1 (rural origin; phylogroup A; resistant to AMP, CTX, FEP, CIP, GEN, TOB, AMK; positive for *bla*_SHV-12_; and for *iutA*) and PRL10.1 (rural origin; phylogroup F; serotype O83:H42; ST1485-CC648 (CH231-58); resistant to AMP, CTX, FEP, CIP, SXT; positive for *bla*_SHV-12_ and for *iutA*, *chuA*, *vat, kpsMII*, fulfilling the ExPEC status). 

Three ESBL-producing *K. pneumoniae* were also recovered from three individual dogs (two from urban and one from rural areas). All were positive for the *bla*_CTX-M-15_ and belonged to the ST307. Interestingly, these *K. pneumoniae* isolates were found together with pAmpC-producing *E. coli* (in two dogs) and with one SHV-12 producing *E. coli* (in one dog).

In summary, we have detected a high rate of fecal colonization by ESBL- and pAmpC-producing *Enterobacteriaceae* in urban and rural dogs in Galicia, with no statistically significant differences (20.8% and 19.1%, respectively).

## 3. Discussion

The rate of colonization by MDR bacteria in companion animals has been assessed in several studies, where the prevalence of colonization by ESBL-producing *Enterobacteriaceae* among dogs and cats (including healthy and sick animals) ranged widely—between 3.1% and 55% [11,12]—while the figures reported only for healthy dogs were around 20% [12]. These data match the results obtained here, with a prevalence of ca. 20%, regardless of the origin of the dogs (rural or urban). This is also consistent with data from previous studies, which did not find significant differences with respect to the urbanization level of the dogs analyzed [11]. Colonization by ESBL/pAmpC-producing *Enterobacteriaceae* in companion animals could be related to several factors, including a selective pressure due to previous antibiotic exposure [10] but also to indirect acquisition through raw feeding with meat or carcasses from food-producing animals, which is not uncommon in rural areas. We know that the dogs included in our study had not received any antimicrobial treatment during the previous four weeks before sampling, but, unfortunately, earlier data on antibiotic consumption were not available. A recent study carried out in three European countries (Belgium, Italy, and the Netherlands), found that antimicrobial consumption in companion animals was lower than consumption in food-producing animals. However, the authors reported a high use of WHO critically important antimicrobials, including cefovecin (a third-generation cephalosporin) and quinolones, being that this consumption is higher for dogs than cats [21]. Third and fourth generation cephalosporins, as well as quinolones, have been classified as restricted by the recent categorization of antimicrobials of the European Medicines Agency [22], but they were not prohibited. In a recent survey carried out in Spain, β-lactams and quinolones were the most prescribed antimicrobials in dogs [23]. It is well known that the use of quinolones typically selects ESBL-producing *Enterobacteriaceae* [24]. Considering that 71.4% of the analyzed isolates from our study were resistant to ciprofloxacin, it is tempting to speculate that quinolone exposure in these dogs could have been involved in the selection of ESBL-producing *Enterobacteriaceae*.

The high genetic diversity and ESBL/pAmpC types found in the present study has been previously described within *E. coli* recovered from dogs [17,25], as well as the STs 93, 453, and 770, found here in isolates conforming the ExPEC status [17,25,26]. Five *bla*_CTX-M-14_-carrying isolates recovered in this study belonged to ST770 *Escherichia* clade I. The clade I was considered as a phylogroup of *E. coli* based on the extent of recombination detected with strains belonging to *E. coli sensu stricto* [27]. ST770 is an infrequently reported clone, which has been associated with *bla*_CTX-M-1_ carriage in broilers and poultry in the Netherlands and Switzerland [28,29] and with *bla*_CTX-M-14_ in a patient diagnosed with a urinary tract infection (UTI) in Spain [30]. Also, this clone, harboring *mcr*-1 and *bla*_CTX-M-2_, has been recently recovered from a dog with a UTI in Argentina [31] and associated with pAmpC production, specifically CMY-2, from rooks wintering in Czechia and from broilers in Sweden [32,33]. 

It is of note that in a recent study performed in our region (Galicia) on chicken and turkey meat, we recovered five (5%) ESBL-producing *Escherichia* clade I ST770 (CH116-552) from different samples, all of them positive for the ExPEC status [34]. Furthermore, three of these isolates were O1:H45. In the same study, we recovered ExPEC-positive isolates belonging to the clones O18:H11-A-ST93-CC168 (CH11-neg), O23:H16-B1-ST453-CC86 (CH6-31), and O83:H42-F-ST1485-CC648 (CH231-58). All those isolates from poultry meat were MDR and most of them fluoroquinolone-resistant. As stated above, dogs may acquire antimicrobial resistant *Enterobacteriaceae* via various routes, including raw feeding with chicken meat or carcasses, which is quite common in rural areas. Poultry products can act as a reservoir for human extraintestinal *Enterobacteriaceae* pathogens [34,35,36], so we also hypothesize that such products could be playing a role in their transmission between animals, particularly in rural environments. Importantly, MDR *E. coli* of human clinical origin and characterized as A-ST93 (CH11-neg), B1-ST453 (CH6-31), and F-ST1485 (CH231-58) were also recently reported in the same health area (Galicia) [37,38]. This reinforces the importance of “One-Health” actions against dissemination of antimicrobial resistance.

Other STs detected in *E. coli* from dogs in the present study were ST93 and ST453. *E. coli* ST93 were reported in wild birds in Pakistan, associated with the carriage of bla_CTX-M-15_ [28]; in beef, veal, pork and poultry, associated with *bla*_CTX-M-1_ in Switzerland [28]; and in broiler chickens carrying bla_CTX-M-2_ in Brazil [39]. In addition, ST93 was also associated to the spread of the *mcr*-1 gene in companion animals and retail food in China [40,41]. Regarding infections in humans, *mcr*-1-carrying ST93 *E. coli* was recovered from a patient with bacteremia in Uruguay [42]. *E. coli* ST453 harboring bla_CTX-M-1_ was isolated from pigs and their breeders [43], associated with extraintestinal disease in humans and metritis in cattle in Australia and, carrying *mcr*-1, with wastewater in Japan [44]. 

Despite the fact that the pandemic *E. coli* ST131-B2 was not detected here, eight out of the 39 (20.5%) isolates conformed to the ExPEC status, including one isolate of the global ExPEC lineage F-CC648 belonging to the ST1485 [6]. The phylogroup F together with phylogroup B2 comprise most human clinical ExPEC isolates. Among phylogroup F, the clonal complex 648 (CC648) is a resistance-associated lineage recovered from different sources (human, animal, or environmental) and increasingly associated with extraintestinal pathologies [45]. Importantly, the dog isolate also fulfilled the UPEC status, conjugating in the same isolate a high number of resistance and virulence genes.

In addition to *E. coli* isolates, we have found three dogs carrying CTX-M-15-producing *K. pneumoniae*, which is a major nosocomial pathogen able to persist in many different reservoirs, including not only health care settings but also retail meat, livestock, and wastewater [46,47]. This species belongs to the ESKAPE list and is considered as a pathogen that represents a global threat to human health, especially in hospital environments [1]. Information about ESBL carriage in this species recovered from companion animals, such as dogs, is limited [10]. The ST307 clone of *K. pneumoniae* found in our study is considered a potential high-risk clone for humans and has been associated with different ESBL- and carbapenemase-encoding genes [48,49]. Recently, it has been obtained from sick and healthy dogs in Vila Real, a city in northern Portugal very close to Galicia, carrying *bla*_CTX-M-15_ and *bla*_SHV-28_ [50]. This clone (including CTX-M-15- SHV-28-producing isolates) was also detected in 27% of the poultry meat samples analyzed by Díaz-Jiménez et al. in Galicia [34].

Fortunately, no carbapenemase-producing *Enterobacteriaceae* were recovered among the dogs studied. In a previous work, a 0.6% prevalence of carbapenemase-producing *Enterobacteriaceae* in the fecal microbiota of companion dogs attending a veterinary hospital in the Community of Madrid (Spain) was reported [13]. Probably because carbapenems are not used in veterinary medicine, bacteria resistant to these drugs are less common than ESBL-producing bacteria in companion animals [14].

The present study has limitations, such as its cross-sectional design, which did not allow subsequent follow up of the dogs studied. Moreover, information about risk factors for MDR bacteria colonization/infection was not available. However, we provide data on the prevalence of ESBL- and pAmpC-producing *Enterobacteriaceae* among healthy rural and urban dogs in northwest Spain, including extraintestinal pathogenic *E. coli* lineages, such as CC648, highlighting the potential role of these animals in the transmission to humans of high-risk pathogens and resistance genes. Therefore, within a “One-Health” approach, their surveillance should be a priority line in the fight against antimicrobial resistance.

## 4. Materials and Methods

### 4.1. Sample Collection, Culture, and Bacterial Identification

A total of 179 fresh fecal specimens were collected during May and June 2019 from individual healthy dogs living in rural and urban environments in Galicia, a ca. 29,500 km^2^ region in Northwest Spain. The healthy status of the dogs was established by the veterinary team in charge of the sampling. Sampling was designed to be representative of the entire territory studied. Thus, a total of 43 different geographical areas were screened, selecting dogs from different rural environments of the four provinces of Galicia (A Coruña, Lugo, Ourense, and Pontevedra), as well as from the main cities in the same region. Urban refers to dogs that live in flats with their owners in large or medium-sized towns in Galicia. Their function is as a companion animal (pets) and they do not contact with livestock. In contrast, rural refers to dogs which usually live in rural areas, in smaller towns or villages. But the most important is that the latter are used as guard dogs in farms, or for hunting. Most of these animals are in contact with livestock (poultry, ruminants -bovine and ovine- and porcine) and with wildlife. The sampling size was calculated based on the dog population in Galicia, which according to official data for 2019 (Galician Registry of Identification of Companion Animals, Department of the Environment of the Xunta de Galicia, Spain) is 609,804 dogs. Of these, 147,284 (24.2%) are urban dogs and 462,520 (75.8%) are rural dogs. In the present study, we have sampled 179 dogs, of which 48 are urban (26.8%) and 131 rural (73.2%) [Table S1]. The rural vs. urban proportions of this work were adjusted to the values of the geographic area. In order to avoid biases, in those cases in which the same owner had several dogs or several dogs lived together in the same area, a single sample of a representative individual was collected. Dogs included in the study had not received any antimicrobial treatment during the previous four weeks. Samples were kept refrigerated (4 °C) in sterile swabs until processing in the laboratory within 24 h after sampling. For this, they were plated on Chromagar ESBL (bioMérieux, Marcy l´Étoile, France), Chromid Carba Smart (bioMérieux), Chromagar OXA-48 (bioMérieux), and also on Columbia agar with 5% sheep blood (bioMérieux) used as a growth control. Bacterial isolates growing in selective media were identified by matrix-assisted laser desorption/ionization time-of-flight mass spectrometry (MALDI-TOF/MS, Bruker Daltronics GmbH, Bremen, Germany). 

### 4.2. Antimicrobial Susceptibility Testing and Characterization of Antimicrobial Resistance-Encoding Genes

Antimicrobial susceptibility testing of suspicious enterobacterial colonies growing in selective media was performed by the MicroScan WalkAway system (Beckman Coulter, CA, USA), and the results were interpreted according to the EUCAST 2020 breakpoints [51]. The antibiotics tested included: ampicillin, amoxicillin/clavulanic acid, cefotaxime, cefepime, piperacillin/tazobactam, imipenem, meropenem, ciprofloxacin, trimethoprim/sulfamethoxazole, gentamicin, tobramycin, amikacin, colistin, and tigecycline. 

All isolates were tested for ESBL-encoding genes (*bla*_TEM_, *bla*_SHV_, and *bla*_CTX-M_) and for pAmpCs by PCR amplification followed by sequencing of the positive amplicons using specific primers [Table S2]. Genes encoding plasmid-mediated colistin resistance (*mcr*-1 to *mcr*-5) were also screened as previously described [52].

### 4.3. Characterization of E. coli Isolates: Virulence Traits, Phylogroups, STs and Clonotypes, Serotyping, and PFGE 

All *E. coli* were analyzed by PCR for specific virulence markers, which define the ExPEC status and the UPEC status. Isolates conformed the ExPEC status if they were positive for ≥ two of five determinants, including *papAH* and/or *papC*, *sfa*/*focDE*, *afa*/*draBC*, *kpsM II*, and *iutA* [53], and met the UPEC status if positive for ≥ three of the four genes, including *chuA*, *fyuA*, *vat*, and *yfcV* [54]. Those isolates exhibiting ExPEC and/or UPEC status were also characterized for other extraintestinal virulence factors: *fimAv_MT78_*, *papEF*, *papC*, *cnf1*, *cdtB*, *sat*, *hlyA*, *hlyF*, *iucD*, *iroN*, *kpsM II* (establishing *neu*C-K1, K2, and K5 variants), *kpsM III*, *cvaC*, *iss*, *traT*, *ibeA*, *malX*, *usp*, *tsh*, and *ompT* [Table S3].

The phylogroup of *E. coli* isolates was determined following the scheme of Clermont et al. [27] [Table S4]. Isolates with ExPEC and/or UPEC status were further characterized for their serotypes, clonotypes and STs. Serotyping was established using the method previously described by Guinee et al. [55] with antisera against O (O1 to O185) and H (H1 to H56) antigens. Clonotyping was accomplished by sequencing 469 nucleotides (nt) internal to the *fumC* gene and 489 nt internal to *fimH*, which allowed us to define the CH type [56] [Table S5]. ST assignment for *E. coli* and for *K. pneumoniae* isolates was performed according to the Achtman and the Diancourt MLST schemes, respectively [57,58] [Table S6 and Table S7].

Pulsed-field gel electrophoresis (PFGE) was performed to *E. coli* isolates as previously described using XbaI [34], and the profiles obtained were compared and analyzed by InfoQuest™FP v.4.5 software (Bio-Rad Laboratories). A dendrogram was constructed by the UPGMA (Unweighted Pair Group Method with Arithmetic Mean) method, based on Dice’s similarity coefficient (1.5% band tolerance; 1.5% optimization).

### 4.4. Statistical Analysis

Differences in colonization between urban and rural dogs were analyzed by a two-tailed Fisher’s exact test, with p values of less than 0.05 being considered as statistically significant.

## 5. Conclusions

Our study highlights the potential role of both rural and urban dogs as a reservoir of high-risk *Enterobacteriaceae* clones, such as the CC648 of *E. coli* and antimicrobial resistance traits. Within a One-Health approach, their surveillance should be a priority in the fight against antimicrobial resistance.

## Figures and Tables

**Table 1:** Phenotypic and genotypic characterization of the eight *E. coli* conforming the ExPEC status.

Isolate	Serotype	PG	ST (CC)	^1^ CH	ESBL	^2^ Antimicrobial Resistance	^3^ Virulence-Gene Profile
PRL11.2	O1:H45	clade I	770 (None)	116-552	CTX-M-14	AMP-AMC-CTX-CIP-GTM-TOB	*fimH552 iucD iutA kpsM II-K5 traT malX chuA*
PRL12.2	O1:H45	clade I	770 (None)	116-552	CTX-M-14	AMP-CTX-FEP-CIP-GTM-TOB-AMK	*fimH552 hlyF iucD iutA kpsM II-K5 traT malX chuA*
PRL15.1	O1:H45	clade I	770 (None)	116-552	CTX-M-14	AMP-CTX-FEP-CIP-GTM-TOB-AMK	*fimH552 hlyF iucD iutA kpsM II-K5 traT malX chuA*
PRL109.1	O1:H45	clade I	770 (None)	116-552	CTX-M-14	AMP-CTX-FEP-CIP-GTM-TOB	*fimH552 hlyF iucD iutA kpsM II-K5 traT malX chuA*
PRL186.1	O1:H45	clade I	770 (None)	116-552	CTX-M-14	AMP-CTX-FEP-CIP-GTM-TOB-AMK	*fimH552 hlyF iucD iutA kpsM II-K5 traT malX chuA*
PRL167.1	O18:H11	A	93 (168)	11-neg	SHV-12	AMP-CTX-FEP	*hlyF iucD iutA kpsM II-K5*
PRL10.3	O23:H16	B1	453 (86)	6-31	SHV-12	AMP-CTX-FEP-CIP-GTM-TOB	*fimH31 hlyF iucD iutA iron kpsM II-K5 cvaC traT iss fyuA*
PRL10.1	O83:H42	F	1485 (648)	231-58	SHV-12	AMP-CTX-FEP-CIP- SXT	*fimH48 hlyF iucD iutA iron kpsM II-K5 cvaC traT malX tsh ompT iss chuA vat fyuA yfcV*

^1^ Clonotype (CH) based on the internal 469-nucleotide (nt) and 489-nt sequence of the *fumC* (allele obtained from multilocus sequence typing (MLST) and *fimH* genes, respectively (Weissman et al., 2012): neg when PCR was negative for the 489-nt internal sequence amplification. ^2^ Phenotypic resistance interpreted according to the European Committee on Antimicrobial Susceptibility testing (EUCAST) guidelines: AMP, ampicillin; AMC, amoxicillin/clavulanic acid; CTX, cefotaxime; FEP, cefepime; CIP, ciprofloxacin; SXT, trimethoprim/sulfamethoxazole; GTM, gentamicin; TOB, tobramycin; AMK, amikacin. ^3^ PRL10.1 isolate complied also with the UPEC status. PG, phylogroup; ST, sequence type; CC, clonal complex.

## Supplementary Materials

The following are available online at https://www.mdpi.com/2079-6382/9/8/468/s1, Table S1: dog sampling data; Table S2: primers used for the detection and/or sequencing of *bla*_CTX-M_, *bla*_SHV_, *bla*_TEM_, *bla*_CMY_, and *mcr* genes; Table S3: targets and primers associated with extraintestinal pathogenic *E. coli*; Table S4: targets and primers to determine phylogroups of *E. coli* (Clermont et al., 2013); Table S5: targets and primers used to determine clonotypes; Table S6: targets and primers to determine sequence types by MLST (*E. coli*), Table S7: targets and primers to determine sequence types by MLST (*K. pneumoniae*).
