# Supplementary material for: High Prevalence and Diversity of Cephalosporin-Resistant Enterobacteriaceae Including Extraintestinal Pathogenic E. coli CC648 Lineage in Rural and Urban Dogs in Northwest Spain
Source: Antibiotics (Basel). 2020 Aug 1;9(8):468. doi: 10.3390/antibiotics9080468 (PMC7460362; doi:10.3390/antibiotics9080468)
Supplement: Supplementary file 1 [file antibiotics-09-00468-s001.pdf]

Table S1. Dog sampling data.

Sampling date	Province	Location	Environment	Dog ID	Isolate ID
May 2019	Lugo	BARALLA	Rural	PRL-01	
May 2019	Lugo	BARALLA	Rural	PRL-02	
May 2019	Lugo	BARALLA	Rural	PRL-03	
May 2019	Lugo	BARALLA	Rural	PRL-04	
May 2019	Lugo	PORTOMARÍN	Rural	PRL-05	
May 2019	Lugo	PORTOMARÍN	Rural	PRL-06	
May 2019	Lugo	TABOADA	Rural	PRL-07	
May 2019	Lugo	TABOADA	Rural	PRL-08	
May 2019	Lugo	TABOADA	Rural	PRL-09	
May 2019	Lugo	ESCAIRÓN	Rural	PRL-10	PRL-10-1
					PRL-10-2
					PRL-10-3
May 2019	Lugo	ESCAIRÓN	Rural	PRL-11	PRL-11-1
					PRL-11-2
May 2019	Lugo	ESCAIRÓN	Rural	PRL-12	
					PRL-12-2
May 2019	Ourense	A RÚA	Rural	PRL-13	
May 2019	Ourense	A RÚA	Rural	PRL-14	
May 2019	Ourense	A RÚA	Rural	PRL-15	PRL-15-1
May 2019	Ourense	A RÚA	Rural	PRL-16	
May 2019	Ourense	A RÚA	Rural	PRL-17	
May 2019	Ourense	MONTEDERRAMO	Rural	PRL-18	
May 2019	Ourense	MONTEDERRAMO	Rural	PRL-19	PRL-19-1
May 2019	Ourense	MONTEDERRAMO	Rural	PRL-20	PRL-20-1
May 2019	Ourense	MONTEDERRAMO	Rural	PRL-21	
May 2019	La Coruña	MELIDE	Rural	PRL-22	
May 2019	La Coruña	MELIDE	Rural	PRL-23	PRL-23-1
May 2019	La Coruña	MELIDE	Rural	PRL-24	PRL-24-1
May 2019	La Coruña	MELIDE	Rural	PRL-25	PRL-25-1
May 2019	La Coruña	MELIDE	Rural	PR-26	PRL-26-1
May 2019	La Coruña	MELIDE	Rural	PRL-27	
May 2019	La Coruña	MELIDE	Rural	PRL-28	
May 2019	La Coruña	ARZÚA	Rural	PRL-29	
May 2019	La Coruña	ARZÚA	Rural	PRL-30	PRL-30-1
May 2019	La Coruña	ARZÚA	Rural	PRL-31	PRL-31-1
May 2019	La Coruña	ARZÚA	Urban	PRL-32	
May 2019	La Coruña	ARZÚA	Rural	PRL-33	PRL-33-1
May 2019	La Coruña	ARZÚA	Rural	PRL-34	PRL-34-1
May 2019	Lugo	LUGO	Urban	PRL-35	PRL-35-1
					PRL-35-2

May 2019	Lugo	LUGO	Urban	PRL-36	
May 2019	Lugo	LUGO	Urban	PRL-37	PRL-37-1
May 2019	Lugo	LUGO	Urban	PRL-38	PRL-38-1
May 2019	Lugo	LUGO	Urban	PRL-39	PRL-39-1
May 2019	Lugo	LUGO	Urban	PRL-40	
May 2019	Lugo	PORTOMARÍN	Rural	PRL-41	
May 2019	Lugo	PORTOMARÍN	Rural	PRL-42	
May 2019	Lugo	PORTOMARÍN	Rural	PRL-43	
May 2019	Lugo	PORTOMARÍN	Rural	PRL-44	
May 2019	Lugo	PORTOMARÍN	Rural	PRL-45	
May 2019	Lugo	LUGO	Rural	PRL-46	
May 2019	Lugo	LUGO	Rural	PRL-47	
May 2019	Lugo	LUGO	Rural	PRL-48	
May 2019	Lugo	LUGO	Urban	PRL-49	PRL-49-1
May 2019	Lugo	LUGO	Urban	PRL-50	
May 2019	La Coruña	TOURO	Rural	PRL-51	
May 2019	La Coruña	TOURO	Rural	PRL-52	
May 2019	La Coruña	TOQUES	Rural	PRL-53	
May 2019	La Coruña	VEDRA	Rural	PRL-54	
May 2019	La Coruña	TOURO	Rural	PRL-55	PRL-55-1
May 2019	La Coruña	VEDRA	Rural	PRL-56	
May 2019	La Coruña	PADRÓN	Rural	PRL-57	
May 2019	Pontevedra	CRECENTE	Rural	PRL-58	
May 2019	La Coruña	SAN SADURNIÑO	Rural	PRL-59	
May 2019	La Coruña	PADRÓN	Rural	PRL-60	
May 2019	Pontevedra	BARRO	Rural	PRL-61	
May 2019	Pontevedra	PONTEVEDRA	Rural	PRL-62	
May 2019	La Coruña	TOMIÑO	Rural	PRL-63	
May 2019	La Coruña	VALDOVIÑO	Rural	PRL-64	
May 2019	Pontevedra	PONTEVEDRA	Rural	PRL-65	
May 2019	La Coruña	CEDEIRA	Rural	PRL-66	
May 2019	La Coruña	TOMIÑO	Rural	PRL-67	
May 2019	Pontevedra	PONTEVEDRA	Rural	PRL-68	
May 2019	La Coruña	TOURO	Rural	PRL-69	
May 2019	Pontevedra	TOMIÑO	Rural	PRL-70	PRL-70-1
May 2019	La Coruña	TOQUES	Rural	PRL-71	
May 2019	La Coruña	TOURO	Rural	PRL-72	
May 2019	La Coruña	VALDOVIÑO	Rural	PRL-73	
May 2019	La Coruña	SAN SADURNIÑO	Rural	PRL-74	PRL-74-1
May 2019	La Coruña	SAN SADURNIÑO	Rural	PRL-75	
May 2019	Lugo	SARRIA	Urban	PRL-76	PRL-76-1

					PRL-76-2
May 2019	Lugo	SARRIA	Urban	PRL-77	PRL-77-1
May 2019	Lugo	PARADELA	Rural	PRL-78	
May 2019	Lugo	PARADELA	Rural	PRL-79	
May 2019	Lugo	PARADELA	Rural	PRL-80	
May 2019	Lugo	PARADELA	Rural	PRL-81	
May 2019	Lugo	PARADELA	Rural	PRL-82	
May 2019	Lugo	PARADELA	Rural	PRL-83	
May 2019	Lugo	PARADELA	Rural	PRL-84	
May 2019	Lugo	PARADELA	Rural	PRL-85	
May 2019	Lugo	TABOADA	Rural	PRL-86	
May 2019	Lugo	TABOADA	Rural	PRL-87	
May 2019	Lugo	TABOADA	Rural	PRL-88	
May 2019	Lugo	CERVO	Urban	PRL-89	
May 2019	Lugo	CERVO	Urban	PRL-90	
May 2019	Lugo	CERVO	Urban	PRL-91	
May 2019	Lugo	CERVO	Urban	PRL-92	
May 2019	Lugo	BURELA	Urban	PRL-93	
May 2019	Lugo	BURELA	Urban	PRL-94	
May 2019	Lugo	BURELA	Urban	PRL-95	
May 2019	Lugo	BURELA	Urban	PRL-96	
May 2019	La Coruña	OLEIROS	Urban	PRL-97	
May 2019	La Coruña	OLEIROS	Urban	PRL-98	
May 2019	La Coruña	OLEIROS	Urban	PRL-99	
May 2019	La Coruña	OLEIROS	Urban	PRL-100	
May 2019	La Coruña	OLEIROS	Urban	PRL-101	
May 2019	La Coruña	CULLEREDO	Urban	PRL-102	
May 2019	La Coruña	CULLEREDO	Urban	PRL-103	
May 2019	La Coruña	CULLEREDO	Urban	PRL-104	
May 2019	La Coruña	CULLEREDO	Urban	PRL-105	
May 2019	La Coruña	CULLEREDO	Urban	PRL-106	PRL-106-1
May 2019	La Coruña	CULLEREDO	Urban	PRL-107	
May 2019	La Coruña	CULLEREDO	Urban	PRL-108	
May 2019	La Coruña	CULLEREDO	Urban	PRL-109	PRL-109-1
May 2019	La Coruña	CULLEREDO	Urban	PRL-110	
May 2019	La Coruña	A CORUÑA	Urban	PRL-111	
May 2019	La Coruña	A CORUÑA	Urban	PRL-112	
May 2019	La Coruña	A CORUÑA	Urban	PRL-113	
May 2019	La Coruña	A CORUÑA	Urban	PRL-114	
May 2019	La Coruña	A CORUÑA	Urban	PRL-115	
May 2019	La Coruña	A CORUÑA	Urban	PRL-116	
May 2019	La Coruña	A CORUÑA	Urban	PRL-117	
May 2019	La Coruña	A CORUÑA	Urban	PRL-118	

May 2019	Ourense	QUINTELA DE LEIRADO	Rural	PRL-124	
May 2019	Ourense	QUINTELA DE LEIRADO	Rural	PRL-126	
May 2019	Ourense	CELANOVA	Rural	PRL-130	
May 2019	Ourense	CELANOVA	Rural	PRL-131	
May 2019	Ourense	CELANOVA	Rural	PRL-132	PLR-132-1
May 2019	Ourense	SANDIÁS	Rural	PRL-134	PLR-134-1
May 2019	Ourense	SANDIÁS	Rural	PRL-135	PLR-135-1
May 2019	Ourense	VEREA	Rural	PRL-138	
May 2019	Ourense	VEREA	Rural	PRL-140	
May 2019	Ourense	BALTAR	Rural	PRL-141	
May 2019	Ourense	BALTAR	Rural	PRL-142	
May 2019	Ourense	BALTAR	Rural	PRL-146	
May 2019	Ourense	BALTAR	Rural	PRL-147	
May 2019	Ourense	BALTAR	Rural	PRL-148	
May 2019	Ourense	XINZO DE LIMIA	Rural	PRL-152	
May 2019	Ourense	XINZO DE LIMIA	Rural	PRL-154	
May 2019	Ourense	XINZO DE LIMIA	Rural	PRL-156	
May 2019	Ourense	XINZO DE LIMIA	Rural	PRL-157	
May 2019	Ourense	BALTAR	Rural	PRL-162	
May 2019	Ourense	BALTAR	Rural	PRL-165	
May 2019	Ourense	VILAR DE SANTOS	Rural	PRL-166	
May 2019	Ourense	VILAR DE SANTOS	Rural	PRL-167	PRL-167-1<br>PRL-167-2
May 2019	Ourense	VILAR DE SANTOS	Rural	PRL-170	PRL-170-1<br>PRL-170-2
May 2019	Pontevedra	SALVATERRA DO MIÑO	Rural	PRL-172	
May 2019	Pontevedra	SALVATERRA DO MIÑO	Rural	PRL-173	
May 2019	Pontevedra	SALVATERRA DO MIÑO	Rural	PRL-174	
May 2019	Pontevedra	SALVATERRA DO MIÑO	Rural	PRL-175	
May 2019	Pontevedra	O ROSAL	Rural	PRL-176	
May 2019	Pontevedra	O ROSAL	Rural	PRL-178	
May 2019	Pontevedra	O ROSAL	Rural	PRL-179	
May 2019	Lugo	VICEDO	Rural	PRL-183	
May 2019	Lugo	VIVEIRO	Rural	PRL-184	PRL-184-1
May 2019	Pontevedra	SILLEDA	Rural	PRL-185	
May 2019	Pontevedra	TOMIÑO	Rural	PRL-186	PRL-186-1
May 2019	Pontevedra	TOMIÑO	Rural	PRL-187	
May 2019	Pontevedra	O ROSAL	Rural	PRL-188	
May 2019	Pontevedra	O ROSAL	Rural	PRL-189	
May 2019	Pontevedra	O ROSAL	Rural	PRL-190	
May 2019	Lugo	MONDOÑEDO	Rural	PRL-191	
May 2019	La Coruña	RIBEIRA	Rural	PRL-192	
May 2019	La Coruña	VALDOVIÑO	Rural	PRL-193	

May 2019	La Coruña	ORDES	Rural	PRL-194	
May 2019	La Coruña	RIBEIRA	Rural	PRL-195	
May 2019	La Coruña	RIBEIRA	Rural	PRL-196	
May 2019	La Coruña	NARÓN	Rural	PRL-197	
May 2019	La Coruña	NARÓN	Rural	PRL-198	
May 2019	La Coruña	ORDES	Rural	PRL-199	
May 2019	La Coruña	NARÓN	Rural	PRL-200	
May 2019	Lugo	PORTOMARÍN	Rural	PRL-201	
May 2019	Lugo	PORTOMARÍN	Rural	PRL-202	
May 2019	Lugo	PORTOMARÍN	Rural	PRL-203	
May 2019	Lugo	PORTOMARÍN	Rural	PRL-204	PRL-204-1
May 2019	Lugo	PORTOMARÍN	Rural	PRL-205	
May 2019	Lugo	PORTOMARÍN	Rural	PRL-206	
June 2019	Pontevedra	PONTEVEDRA	Urban	PRL-207	PRL-207-1
June 2019	Pontevedra	PONTEVEDRA	Urban	PRL-208	
June 2019	Pontevedra	PONTEVEDRA	Urban	PRL-209	
June 2019	Pontevedra	PONTEVEDRA	Urban	PRL-210	
June 2019	Pontevedra	PONTEVEDRA	Urban	PRL-211	
June 2019	Pontevedra	PONTEVEDRA	Urban	PRL-212	
June 2019	Pontevedra	PONTEVEDRA	Urban	PRL-213	

Table S2. Primers used for the detection and/or sequencing of *bla*$_{CTX-M}$, *bla*$_{SHV}$, *bla*$_{TEM}$, *bla*$_{CMY}$, and *mcr* genes.

**Target**	***Primers***	**Nucleotide sequence (5´- 3´)**	**Size (bp)**	**Reference**
bla$_{CTX-M}$	CTX-C3	ATGTGCAGCACCAGTAAAGTGATG	542	Mora et al., 2013
	CTX-C4	ACCGCGATATCGTTGGTGGTGCC		
bla$_{CTX-M-group 1}$	M13U	GGTTAAAAAATCACTGCGTC	863	Saladin et al., 2002
	M13L	TTGGTGACGATTTTAGCCGC		
bla$_{CTX-M- group 1}$	[b]CTX-15-F1	GAAGCTAATAAAAAACACACGTGG	1044-1123	Mora et al., 2013
	[b]CTX-15-R	GTATGCGCAAGCGCAGGTGG		
bla$_{CTX-M- group 9}$	[a]CTX M9 -1F/4R	TGGTGACAAAGAGAGTGCAACG	874	Paauw et al., 2006
	[a]CTX M9 - 4R	TCACAGCCCTTCGGCGAT		
bla$_{SHV}$	SHV-F2	TTGTCGCTTCTTTACTCGCC	879	Mora et al., 2013
	SHV-R2	CCCGGCGATTTGCTGATTTCGC		
bla$_{SHV}$	[b]SHV-1	GGGTTATTCTTATTTGTCGC	930	Rasheed et al., 1997
	[b]SHV-2	TTAGCGTTGCCAGTGCTC		
bla$_{TEM}$	[a]TEM-1F	ATGAGTATTCAACATTTCCG	868	Rasheed et al., 1997
	[a]TEM-1R	CTGACAGTTACCAATGCT TA		
LAT-1 a LAT-4, CMY-2 a CMY-7, BIL-1	CITMF	TGGCCAGAACTGACAGGCAAA	462	Pérez-Pérez & Hanson, 2002
	CITMR	TTTCTCCTGAACGTGGCTGGC		
CMY-2	[b]CMY-2F	AACACACTGATTGCGTCTGAC	1226	Pérez-Pérez & Hanson, 2002
	[b]CMY-2R	CTGGGCCTCATCGTCAGTTA		
mcr-1	mcr1_320bp_fw	AGTCCGTTTGTTCTTGTGGC	320	Rebelo et al., 2018
	mcr1_320bp_rev	AGATCCTTGGTCTCGGCTTG		
	mcr-2 IR	AGATGGTATTGTTGGTTGCTG		
mcr-2	mcr2_700bp_fw	CAAGTGTGTTGGTCGCAGTT	715	Rebelo et al., 2018
	mcr2_700bp_rev	TCTAGCCCGACAAGCATACC		
mcr-3	mcr3_900bp_fw	AAATAAAAATTGTTCCGCTTATG	929	Rebelo et al., 2018
	mcr3_900bp_rev	AATGGAGATCCCCGTTTTT		
mcr-4	mcr4_1100bp_fw	TCACTTTCATCACTGCGTTG	1116	Rebelo et al., 2018
	mcr4_1100bp_rev	TTGGTCCATGACTACCAATG		
mcr-5	MCR5_FW	ATGCGGTTGTCTGCATTTATC	1644	Borowiak et al., 2017
	MCR5_RV	TCATTGTGGTTGTCCTTTTCTG		

[a]Primers used for amplification and sequencing. [b]Primers used for sequencing.

Table S3. Targets and primers associated with extraintestinal pathogenic *E. coli.*

Target	*Primers*	Nucleotide sequence (5´- 3´)	Size (bp)	Reference
fimH	FimH F	TGCAGAACGGATAAGCCGTGG	508	Johnson & Stell, 2000
	FimH R	GCAGTCACCTGCCCTCCGGTA		
fimAvMT78	fimA215	ACTTTAGGATGAGTACTG	266	Marc & Dho-Moulin, 1996
	fimA201	TCTGGCTGATACTACACC		
papAH	papA-F	ATGGCAGTGGTGTCTTTTGGTG	720	Johnson & Stell, 2000
	papA-R	CGTCCCACCATACGTGCTCTTC		
papEF	PapEF F	GCAACAGCAACGCTGGTTGCATCAT	336	Yamamoto et al., 1995
	PapEF R	AGAGAGAGCCACTCTTATACGGACA		
PapG1	pap-I F	TTAGCTGGATGGCACAATG	335	Mora et al., 2013
	pap-I R	TTGTCCATGTATCCCATTCAT		
papGII	Pap-II f	GGGCATTGCTACGGTAACCTG	545	Mora et al., 2013
	Pap-II r	CGCTATTAATAGACAGATCACC		
papGIII	Pap-III f	CGGCAACTTTAAGCTATGTG	720	Mora et al., 2013
	Pap-III r	TGTACCATCTCATCGTTGTCTC		
papC	Forward	GTGGCAGTATGAGTAATGACCGTTA	205	Johnson et al., 2015
	Reverse	ATATCCTTTCTGCAGGGATGCAATA		
[a]*papAH*	papA-F	ATGGCAGTGGTGTCTTTTGGTG	720	Johnson & Stell, 2000
	papA-R	CGTCCCACCATACGTGCTCTTC		
[a]*sfa/focDE*	sfa 1	CTCCGGAGAACTGGGTGCATCTTAC	410	Le Bouguenec et al., 1992
	sfa 2	CGGAGGAGTAATTACAAACCTGGCA		
[b]*yfcV*	yfcV-F	ACATGGAGACCACGTTCACC	292	Spurbeck et al., 2012
	YfcV-R	GTAATCTGGAATGTGGTCAGG		
afaFM955459	Afa-025F	GAGTCACGGCAGTCGCGGCGG	207	Blanco et al., 2009
	Afa-025R	TTCACCGGCGACCAGCCATCTCC		
[a]*afa/draBC*	afa1	GCTGGGCAGCAAACTGATAACTCTC	750	Le Bouguenec et al., 1992
	afa2	CATCAAGCTGTTTGTTCGTCCGCCG		
cnf 1	CNF1-F2	CAGGAGGTACTTAGCAGCGT	468	Mora et al., 2013
	CNF1-RC	TAATTTTGGGTTTGTATC		
cdtB	cdt-s1	GAAAGTAAATGGAATATAAATGTCCG	466	Tóth et al., 2003
	cdt-as1	AAATCTCCTGCAATCATCCAGTTA		
	cdt-s2	GAAAATAAATGGAACACACATGTCCG		
	cdt-as2	AAATCACCAAGAATCATCCAGTTA		
hlyA	hly f	AACAAGGATAAGCACTGTTCTGGCT	1177	Yamamoto et al., 1995
	hly r	ACCATATAAGCGGTCATTCCCGTCA		
hlyF	Forward	TCGTTTAGGGTGCTTACCTTCAAC	444	Morales et al., 2004
	Reverse	TTTGGCGGTTTAGGCATTCC		
sat	SatF	GCAGCTACCGCAATAGGAGGT	937	Johnson et al., 2003
	SatR	CATTCAGAGTACCGGGGCCTA		
[b]*vat*	vat-F	TCAGGACACGTTCAGGCATTCAGT	1100	Spurbeck et al., 2012a
	vat-R	GGCCAGAACATTTGCTCCCTTGTT		
*iro*N	Ironec-f	AAGTCAAAGCAGGGGTTGCCCG	665	Johnson et al., 2000
	Ironec-r	GACGCCGACATTAAGACGCAG		
[b]*fyuA*	fyuA-F	GTAAACAATCTTCCCGCTCGGCAT	850	Spurbeck et al., 2012
	fyuA-R	TGACGATTAACGAACCGGAAGGGA		
[b]*chuA*	ChuA-F	CTGAAACCATGACCGTTACG	652	Spurbeck et al., 2012
	ChuA-R	TTGTAGTAACGCACTAAACC		
*iuc*D	Aer f	TACCGGATTGTCATATGCAGACCGT	602	Yamamoto et al., 1995
	Aer r	AATATCTTCCTCCAGTCCGGAGAAG		
[a]*iut*A	aer-851f	GGCTGGACATCATGGGAACTGG	301	Johnson et al., 1997
	aer-1152r	CGTCGGGAACGGGTAGAATCG		
[a]*kpsM II*	KpsII f	GCGCATTTGCTGATACTGTTG	272	Johnson & Stell, 2000
	KpsII r	CATCCAGACGATAAGCATGAGCA		
kpsM II-K2	kpsII f	GCGCATTTGCTGATACTGTTG	570	(Johnson and O'Bryan, 2004)
	KpsII-K2r	AGGTAGTTCAGACTCACACCT		
kpsM II-K5	K5 f	CAGTATCAGCAATCGTTCTGTA	159	Johnson and Stell, 2000
	kpsII r	CATCCAGACGATAAGCATGAGCA		
neuC-K1	neu1	AGGTGAAAAGCCTGGTAGTGTG	676	Moulin-Schouleur et al., 2006
	neu2	GGTGGTACATCCCGGGATGTC		
kpsM III	kps III f	TCCTCTTGCTACTATTCCCCCT	392	Johnson & Stell, 2000
	kps III r	AGGCGTATCCATCCCTCCTAAC		
cvaC	CoIV-Cf	CACACACAAACGGGAGCTGTT	680	Johnson & Stell, 2000
	CoIV-Cr	CTTCCCGCAGCATAGTTCCAT		
ibeA	Ibe10 f	AGGCAGGTGTGCGCCGCGTAC	170	Johnson & Stell, 2000
	Ibe10 r	TGGTGCTCCGGCAAACCATGC		
iss	is-f	CAGCAACCCGAACCACTTGATG	323	Johnson et al., 2008b
	is-r	AGCATTGCCAGAGCGGCAGAA		
malX	MALX-F	GCATGAGCAGTGCGATACATCGC	828	Mora et al., 2013
	MALX-R	AGGGCTGGGAAGTGGTTTAGCC		
ompT	Forward	ATCTAGCCGAAGAAGGAGGC	559	Johnson et al., 2015
	Reverse	CCCGGGTCATAGTGTTCATC		
traT	TraTf	GGTGTGGTGCGATGAGCACAG	290	Johnson & Stell, 2000
	TraTr	CACGGTTCAGCCATCCCTGAG		
tsh	tsh03	GGTGGTGCACTGGAGTGG	640	Dozois et al., 2000
	tsh15	AGTCCAGCGTGATAGTGG		
usp	usp-f	ACATTCACGGCAAGCCTCAG	440	Bauer et al., 2002
	usp-r	AGCGAGTTCCTGGTGAAAGC		
rfbO25b	rfbO25b.r	TGCTATTCATTATGCGCAGC	300	Clermont et al., 2008
	rfb.1bis	ATACCGACGACGCCGATCTG		

[a]Virulence markers screened to assess the extraintestinal pathogenic *E. coli* status (ExPEC status). [b]Virulence markers screened to assess the uropathogenic *E. coli* status (UPEC status).

Table S4. Targets and primers to determine phylogroups of *E. coli* (Clermont et al., 2013).

Target	*Primers*	Nucleotide sequence (5´- 3´)	Size (bp)	Reference
chuA	ChuA.1b	ATGGTACCGGACGAACCAAC	288	Clermont et al., 2013
	ChuA.2b	TGCCGCCAGTACCAAAGACA		Clermont et al., 2000
yjaA	YjaA.1b	CAAACGTGAAGTGTCAGGAG	211	Clermont et al., 2013
	YjaA.2b	AATGCGTTCCTCAACCTGTG		
TSPE4.C2	TspE4C2.1b	CACTATTCGTAAGGTCATCC	152	
	TspE4C2.2b	AGTTTATCGCTGCGGGTCGC		
arpA	AceK.f	AACGCTATTCGCCAGCTTGC	400	
	ArpA1.r	TCTCCCCATACCGTACGCTA		
TrpAgpC (filogrupo C)	trpAgpC.1	AGTTTTATGCCCAGTGCGAG	219	Lescat et al., 2013
	trpAgpC.2	TCTGCGCCGGTCACGCCC		
*arp*A (filogrupo E)	ArpAgpE.f	GATTCCATCTTGTCAAAATATGCC	301	Lescat et al., 2013
	ArpAgpE.r	GAAAAGAAAAAGAATTCCCAAGAG		
trpA	trpBA.f	CGGCGATAAAGACATCTTCAC	489	Clermont et al., 2008
	trpBA.r	GCAACGCGGCCTGGCGGAAG		

Table S5. Targets and primers used to determine clonotypes.

Target	*Primers*	Nucleotide sequence (5´- 3´)	Size (bp)	Reference
fimH	fimH-wf	CACTCAGGGAACCATTCAGGCA	469	Weissman et al., 2012
	fimH-wr	CTTATTGATAAACAAAAGTCAC		
	fumCR	GTACGCAGCGAAAAAGATTC		
fumC	fumCF	TCACAGGTCGCCAGCGCTTC	469	Wirth et al., 2006
	fumCR	GTACGCAGCGAAAAAGATTC		

Table S6. Targets and primers to determine sequence types by multilocus sequence typing (MLST) (*E. coli*).

Achtman seven-locus scheme for *E. coli*				
Target	*Primers*	Nucleotide sequence (5´- 3´)	Size (bp)	Reference
adk	adkF	ATTCTGCTTGGCGCTCCGGG	536	Wirth et al., 2006
	adkR	CCGTCAACTTTCGCGTATTT		
fumC	fumCF	TCACAGGTCGCCAGCGCTTC	469	Wirth et al., 2006
	fumCR	GTACGCAGCGAAAAAGATTC		
gyrB	gyrBF	TCGGCGACACGGATGACGGC	460	Wirth et al., 2006
	gyrBR	ATCAGGCCTTCACGCGCATC		
icd	icdF	ATGGAAAGTAAAGTAGTTGTTCCGGCACA	518	Wirth et al., 2006
	icdR	GGACGCAGCAGGATCTGTT		
mdh	mdhF	ATGAAAGTCGCAGTCCTCGGCGCTGCTGGCGG	452	Wirth et al., 2006
	mdhR	TTAACGAACTCCTGCCCCAGAGCGATATCTTTCTT		
purA	purAF	CGCGCTGATGAAAGAGATGA	478	Wirth et al., 2006
	purAR	CATACGGTAAGCCACGCAGA		
recA	recAR1	AGCGTGAAGGTAAAACCTGTG	510	Wirth et al., 2006
	recAF1	ACCTTTGTAGCTGTACCACG		

Table S7. Targets and primers to determine sequence types by MLST (*Klebsiella pneumoniae*).

Institute Pasteur MLST for *Klebsiella pneumoniae*				
Target	*Primers*	Nucleotide sequence (5´- 3´)	Size (bp)	Reference
rpoB	Vic3	GGCGAAATGGCWGAGAACCA	501	Diancourt et al., 2005
	Vic2	GAGTCTTCGAAGTTGTAACC		
gapA	gapA173	TGAAATATGACTCCACTCACGG	450	

	gapA181	CTTCAGAAGCGGCTTTGATGGCTT		Diancourt et al., 2005
mdh	mdh130	CCCAACTCGCTTCAGGTTCAG	477	Diancourt et al., 2005
	mdh867	CCGTTTTTCCCCAGCAGCAG		
pgi	pgi1F	GAGAAAAACCTGCCTGTACTGCTGGC	432	Diancourt et al., 2005
	pgi1R	CGCGCCACGCTTTATAGCGGTTAAT		
phoE	phoE604.1	ACCTACCGCAACACCGACTTCTTCGG	420	Diancourt et al., 2005
	phoE604.2	TGATCAGAACTGGTAGGTGAT		
infB	infB1F	CTCGCTGCTGGACTATATTCG	318	Diancourt et al., 2005
	infB1R	CGCTTTCAGCTCAAGAACTTC		
tonB	tonB1F	CTTTATACCTCGGTACATCAGGTT	414	Diancourt et al., 2005
	tonB2R	ATTCGCCGGCTGRGCRGAGAG		
For the seven targets	aPrimer oF	GTTTTCCCAGTCACGACGTTGTA		Diancourt et al., 2005
	aPrimer oR	TTGTGAGCGGATAACAATTTC		

References:

1. Bauer, R.J., Zhang, L., Foxman, B., Siitonen, A., Jantunen, M.E., Saxen, H., Marrs, C.F., 2002. Molecular Epidemiology of 3 Putative Virulence Genes for *Escherichia coli* Urinary Tract Infection— *usp, iha,* and *iroN* *E. coli*. J. Infect. Dis. 185, 1521–1524. doi:10.1086/340206
2. Blanco, M., Alonso, M.P., Nicolas-Chanoine, M.-H., Dahbi, G., Mora, A., Blanco, J.E., López, C., Cortés, P., Llagostera, M., Leflon-Guibout, V., Puentes, B., Mamani, R., Herrera, A., Coira, M.A., García-Garrote, F., Pita, J.M., Blanco, J., 2009. Molecular epidemiology of Escherichia coli producing extended-spectrum β-lactamases in Lugo (Spain): dissemination of clone O25b:H4-ST131 producing CTX-M-15. J. Antimicrob. Chemother. 63, 1135–1141. doi:10.1093/jac/dkp122
3. Borowiak, M., Fischer, J., Hammerl, J.A., Hendriksen, R.S., Szabo, I., Malorny, B., 2017. Identification of a novel transposon-associated phosphoethanolamine transferase gene, mcr-5, conferring colistin resistance in d-tartrate fermenting Salmonella enterica subsp. enterica serovar Paratyphi B. J. Antimicrob. Chemother. 72, 3317–3324. doi:10.1093/jac/dkx327
4. Clermont, O., Bonacorsi, S., Bingen, E., 2000. Rapid and simple determination of the Escherichia coli phylogenetic group. Appl. Environ. Microbiol. 66, 4555–8.
5. Clermont, O., Christenson, J.K., Denamur, E., Gordon, D.M., 2013. The Clermont Escherichia coli phylo-typing method revisited: Improvement of specificity and detection of new phylo-groups. Environ. Microbiol. Rep. doi:10.1111/1758-2229.12019
6. Clermont, O., Lavollay, M., Vimont, S., Deschamps, C., Forestier, C., Branger, C., Denamur, E., Arlet, G., 2008. The CTX-M-15-producing Escherichia coli diffusing clone belongs to a highly virulent B2 phylogenetic subgroup. J. Antimicrob. Chemother. 61, 1024–8. doi:10.1093/jac/dkn084
7. Diancourt, L., Passet, V., Verhoef, J., Grimont, P.A.D., Brisse, S., 2005. Multilocus sequence typing of Klebsiella pneumoniae nosocomial isolates. J. Clin. Microbiol. 43, 4178–4182. doi:10.1128/JCM.43.8.4178-4182.2005
8. Dozois, C.M., Dho-Moulin, M., Brée, A., Fairbrother, J.M., Desautels, C., Curtiss, R., 2000. Relationship between the Tsh autotransporter and pathogenicity of avian Escherichia coli and localization and analysis of the Tsh genetic region. Infect. Immun. 68, 4145–54.
9. Johnson, J.R., Gajewski, A., Lesse, A.J., Russo, T.A., 2003. Extraintestinal pathogenic Escherichia coli as a cause of invasive nonurinary infections. J. Clin. Microbiol. 41, 5798–802.
10. Johnson, J.R., O'Bryan, T.T., 2004. Detection of the Escherichia coli group 2 polysaccharide capsule synthesis Gene kpsM by a rapid and specific PCR-based assay. J. Clin. Microbiol. 42, 1773–6.
11. Johnson, J.R., Porter, S., Johnston, B., Kuskowski, M.A., Spurbeck, R.R., Mobley, H.L.T., Williamson, D.A., 2015. Host Characteristics and Bacterial Traits Predict Experimental Virulence for Escherichia coli Bloodstream Isolates From Patients With Urosepsis. Open forum Infect. Dis. 2, ofv083. doi:10.1093/ofid/ofv083
12. Johnson, J.R., Russo, T.A., Tarr, P.I., Carlino, U., Bilge, S.S., Vary, J.C., Stell, A.L., 2000. Molecular epidemiological and phylogenetic associations of two novel putative virulence genes, iha and iroN(E. coli), among Escherichia coli isolates from patients with urosepsis. Infect. Immun. 68, 3040–7.
13. Johnson, J.R., Stapleton, A.E., Russo, T.A., Scheutz, F., Brown, J.J., Maslow, J.N., 1997. Characteristics and prevalence within serogroup O4 of a J96-like clonal group of uropathogenic Escherichia coli O4:H5 containing the class I and class III alleles of papG. Infect. Immun. 65, 2153–9.
14. Johnson, J.R., Stell, A.L., 2000. Extended virulence genotypes of Escherichia coli strains from patients with urosepsis in relation to phylogeny and host compromise. J. Infect. Dis. 181, 261–72. doi:10.1086/315217

15. Johnson, T.J., Wannemuehler, Y.M., Nolan, L.K., 2008. Evolution of the iss Gene in Escherichia coli. Appl. Environ. Microbiol. 74, 2360–2369. doi:10.1128/AEM.02634-07
16. Le Bouguenec, C., Archambaud, M., Labigne, A., 1992. Rapid and specific detection of the pap, afa, and sfa adhesin-encoding operons in uropathogenic Escherichia coli strains by polymerase chain reaction. J. Clin. Microbiol. 30, 1189–93.
17. Lescat, M., Clermont, O., Woerther, P.L., Glodt, J., Dion, S., Skurnik, D., Djossou, F., Dupont, C., Perroz, G., Picard, B., Catzeflis, F., Andremont, A., Denamur, E., 2013. Commensal Escherichia coli strains in Guiana reveal a high genetic diversity with host-dependant population structure. Environ. Microbiol. Rep. 5, 49–57. doi:10.1111/j.1758-2229.2012.00374.x
18. Marc, D., Dho-Moulin, M., 1996. Analysis of the fim cluster of an avian O2 strain of Escherichia coli: serogroup-specific sites within fimA and nucleotide sequence of fimI. J. Med. Microbiol. 44, 444–52. doi:10.1099/00222615-44-6-444
19. Mora, A., Viso, S., López, C., Alonso, M.P., García-Garrote, F., Dabhi, G., Mamani, R., Herrera, A., Marzoa, J., Blanco, M., Blanco, J.E., Moulin-Schouleur, M., Schouler, C., Blanco, J., 2013. Poultry as reservoir for extraintestinal pathogenic Escherichia coli O45:K1:H7-B2-ST95 in humans. Vet. Microbiol. 167, 506–12. doi:10.1016/j.vetmic.2013.08.007
20. Morales, C., Lee, M.D., Hofacre, C., Maurer, J.J., 2004. Detection of a novel virulence gene and a Salmonella virulence homologue among Escherichia coli isolated from broiler chickens. Foodborne Pathog. Dis. 1, 160–5. doi:10.1089/fpd.2004.1.160
21. Moulin-Schouleur, M., Schouler, C., Tailliez, P., Kao, M.-R., Brée, A., Germon, P., Oswald, E., Mainil, J., Blanco, M., Blanco, J., 2006. Common virulence factors and genetic relationships between O18:K1:H7 Escherichia coli isolates of human and avian origin. J. Clin. Microbiol. 44, 3484–92. doi:10.1128/JCM.00548-06
22. Paauw, A., Fluit, A.C., Verhoef, J., Leverstein-Van Hall, M.A., 2006. Enterobacter cloacae outbreak and emergence of quinolone resistance gene in Dutch Hospital. Emerg. Infect. Dis. 12, 807–812. doi:10.3201/eid1205.050910
23. Pérez-Pérez, F.J., Hanson, N.D., 2002. Detection of plasmid-mediated AmpC beta-lactamase genes in clinical isolates by using multiplex PCR. J. Clin. Microbiol. 40, 2153–62.
24. Rasheed, J.K., Jay, C., Metchock, B., Berkowitz, F., Weigel, L., Crellin, J., Steward, C., Hill, B., Medeiros, A.A., Tenover, F.C., 1997. Evolution of extended-spectrum beta-lactam resistance (SHV-8) in a strain of Escherichia coli during multiple episodes of bacteremia. Antimicrob. Agents Chemother. 41, 647–53.
25. Rebelo, A.R., Bortolaia, V., Kjeldgaard, J.S., Pedersen, S.K., Leekitcharoenphon, P., Hansen, I.M., Guerra, B., Malorny, B., Borowiak, M., Hammerl, J.A., Battisti, A., Franco, A., Alba, P., Perrin-Guyomard, A., Granier, S.A., De Frutos Escobar, C., Malhotra-Kumar, S., Villa, L., Carattoli, A., Hendriksen, R.S., 2018. Multiplex PCR for detection of plasmid-mediated colistin resistance determinants, mcr-1, mcr-2, mcr-3, mcr-4 and mcr-5 for surveillance purposes. Euro Surveill. 23. doi:10.2807/1560-7917.ES.2018.23.6.17-00672
26. Saladin, M., Cao, V.T.B., Lambert, T., Donay, J.-L., Herrmann, J.-L., Ould-Hocine, Z., Verdet, C., Delisle, F., Philippon, A., Arlet, G., 2002. Diversity of CTX-M beta-lactamases and their promoter regions from Enterobacteriaceae isolated in three Parisian hospitals. FEMS Microbiol. Lett. 209, 161–8. doi:10.1111/j.1574-6968.2002.tb11126.x
27. Spurbeck, R.R., Dinh, P.C., Walk, S.T., Stapleton, A.E., Hooton, T.M., Nolan, L.K., Kim, K.S., Johnson, J.R., Mobley, H.L.T., 2012. Escherichia coli isolates that carry vat, fyuA, chuA, and yfcV efficiently colonize the urinary tract. Infect. Immun. 80, 4115–22. doi:10.1128/IAI.00752-12
28. Tóth, I., Hérault, F., Beutin, L., Oswald, E., 2003. Production of cytolethal distending toxins by pathogenic Escherichia coli strains isolated from human and animal sources: establishment of the existence of a new cdt variant (Type IV). J. Clin. Microbiol. 41, 4285–91.
29. Weissman, S.J., Johnson, J.R., Tchesnokova, V., Billig, M., Dykhuizen, D., Riddell, K., Rogers, P., Qin, X., Butler-Wu, S., Cookson, B.T., Fang, F.C., Scholes, D., Chattopadhyay, S., Sokurenko, E., 2012. High-Resolution Two-Locus Clonal Typing of Extraintestinal Pathogenic Escherichia coli. doi:10.1128/AEM.06663-11
30. Wirth, T., Falush, D., Lan, R., Colles, F., Mensa, P., Wieler, L.H., Karch, H., Reeves, P.R., Maiden, M.C.J., Ochman, H., Achtman, M., 2006. Sex and virulence in Escherichia coli : an evolutionary perspective. Mol. Microbiol. 60, 1136–1151. doi:10.1111/j.1365-2958.2006.05172.x

31. Yamamoto, S., Terai, A., Yuri, K., Kurazono, H., Takeda, Y., Yoshida, O., 1995. Detection of urovirulence factors in Escherichia coli by multiplex polymerase chain reaction. FEMS Immunol. Med. Microbiol. 12, 85–90. doi:10.1111/j.1574-695X.1995.tb00179.x
